# MicroRNA-19a acts as a prognostic marker and promotes prostate cancer progression via inhibiting VPS37A expression

**DOI:** 10.18632/oncotarget.23026

**Published:** 2017-12-06

**Authors:** Fangqiu Fu, Xuechao Wan, Dan Wang, Zhe Kong, Yalong Zhang, Wenhua Huang, Chenji Wang, Hai Wu, Yao Li

**Affiliations:** ^1^ Obstetrics and Gynecology Hospital, State Key Laboratory of Genetic Engineering, School of Life Science, Fudan University, Shanghai 200433, PR China; ^2^ Key Laboratory of Reproduction Regulation of NPFPC, Fudan University, Shanghai 200433, PR China

**Keywords:** microRNA-19a, VPS37A, prostate cancer, tumor progression, prognostic marker

## Abstract

Prostate cancer (PCa) is a leading cause of cancer-related deaths among males worldwide. However, the molecular mechanisms underlying the progression of PCa remain unclear. Despite several reported miRNAs in prostate cancer, these reports lacked system-level identification of differentially expressed miRNAs in large sample size. Moreover, it's still largely unknown how miRNAs result in tumorigenesis and progression of PCa. Therefore, by analyzing three public databases, we identified 16 upregulated miRNAs and 13 downregulated miRNAs, and validated miR-19a was one of the most upregulated miRNAs using qRT-PCR. The dual-luciferase reporter assays indicated VPS37A was a potential target of miR-19a. Functional assays revealed miR-19a served as an oncogene by inhibiting VPS37A. Notably, a significant inverse correlation of miR-19a and VPS37A expression was observed in PCa specimens. Moreover, miR-19a-high and VPS37A-low phenotypes were associated with poor prognosis with biochemical recurrence-free probability. In this study, we confirmed the oncogenic role of miR-19a via targeting VPS37A in PCa, identifying miR-19a and VPS37A as diagnosis and therapeutic biomarkers for PCa.

## INTRODUCTION

Prostate cancer (PCa) is the one of the most common cancers in males worldwide [1] and accounts for more than one-quarter of new diagnostic cases in America [2]. Although initially slow growing, prostate cancer can progress rapidly, resulting in systemic metastasis [3, 4]. Unfortunately, the molecular mechanisms underlying the progression of PCa remains unclear. The other challenge in PCa treatment is the lack of accurate biomarkers for early diagnosis. Prostate-specific antigen (PSA) was currently main non-invasive biomarker of PCa [5]. However, many non-cancer conditions such as benign prostatic hyperplasia could also induced the rising of PSA levels [6]. Thus, there is an urgent need to explore key regulators of tumor progression and identify potential diagnostic targets for PCa.

MicroRNAs, which is a class of non-coding RNA with 18–25 nucleotides in length, are reported to act as a post-transcriptional regulator by binding to 3′-untranslated region(3′-UTR) of target mRNAs [7–9]. Recently reports have shown microRNAs could potentially regulate cell differentiation and development, metabolism, proliferation, apoptosis and tumorigenesis [10–12]. Many miRNAs are reported to be aberrantly expressed in cancers and can play either tumor-promoting or tumor-suppressing effects [13, 14]. More importantly, the miRNA therapy is proved effective against tumor progression [15]. In PCa, a bit of microRNAs are identified as oncogenes or tumor suppressor such as miR-27a [16], miR-135a [17], and miR-744 [18]. However, these reports lacked systems-level identification of differentially expressed miRNAs in large sample size in PCa.

Here, we profiled the miRNA expression in three public databases (TCGA, GSE21306 and GSE76260) and identified 16 upregulated miRNAs and 13 downregulated miRNAs. Among them, miR-19a was one of the most upregulated miRNAs. We also performed experiments to explore the roles of miR-19a in regulating cell proliferation, cell cycle, cell migration and apoptosis. Mechanistically, we found VPS37A (Vacuolar Protein Sorting 37 homolog A), the direct target of miR-19a, mediated the regulation of miR-19a in PCa progression. Thus, we demonstrated miR-19a was up-regulated in PCa specimens for the first time and it played a key role in PCa unravelling new diagnosis and therapeutic opportunities.

## RESULTS

### Identification of miRNAs aberrantly expressed in prostate cancers

**Figure 1:**
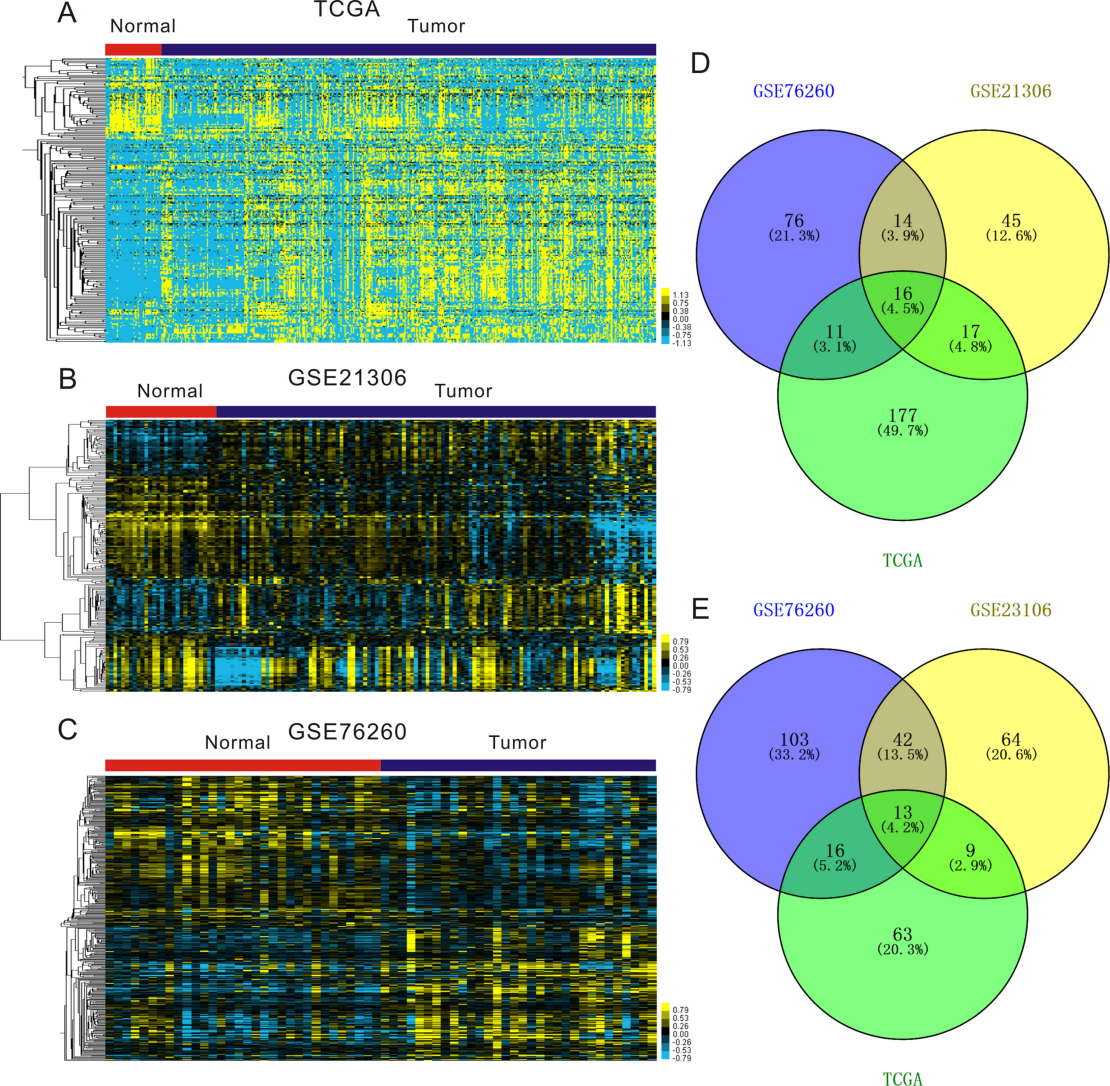
Expression profiles of miRNAs in prostate cancer specimens compared with normal prostate tissues (**A**–**C**) Heatmaps for aberrantly expressed in prostate cancer in publicly available gene expression data TCGA (A), GSE21360 (B) and GSE76260 (C). Samples are in columns and miRNAs are in rows. (**D**–**E**) Venn diagrams for miRNAs whose expression are significantly up-regulated (D) and down-regulated (E) in TCGA, GSE21360 and GSE76260.

In order to better understand the role of miRNAs in PCa progression, we first aimed to identify the significantly differentially expressed miRNAs in PCa using three public databases: TCGA [19], GSE21036 [20] and GSE76260 (Figure 1A–1C). We found 16 significantly up-regulated miRNAs (including hsa-miR-32, hsa-miR-19a, hsa-miR-18b, hsa-miR-96, hsa-miR-183, hsa-miR-130b, hsa-miR-182, hsa-miR-18a, hsa-miR-375, hsa-miR-106a, hsa-miR-106b, hsa-miR-425, hsa-miR-17, hsa-miR-25, hsa-miR-93, and hsa-miR-200c) (Figure 1D and Supplementary Table 1), and 13 significantly down-regulated miRNAs (including hsa-miR-100, hsa-miR-23a, hsa-miR-29a, hsa-miR-23b, hsa-miR-27b, hsa-miR-378, hsa-miR-221, hsa-miR-222, hsa-miR-152, hsa-miR-205, hsa-miR-379, hsa-miR-136, and hsa-miR-452) (Figure 1E and Supplementary Table 2). In present study, we are primarily focusing on the up-regulated miRNAs as putative biomarkers in prostate tumor compared with normal tissues. Among 16 up-regulated miRNAs in PCa, miR-19a was one of the most upregulated miRNAs and selected for further study. Clustering analysis was subsequently performed for all abnormally expressed miRNAs in databases.

### MiR-19a is upregulated in prostate cancer tissues

**Figure 2:**
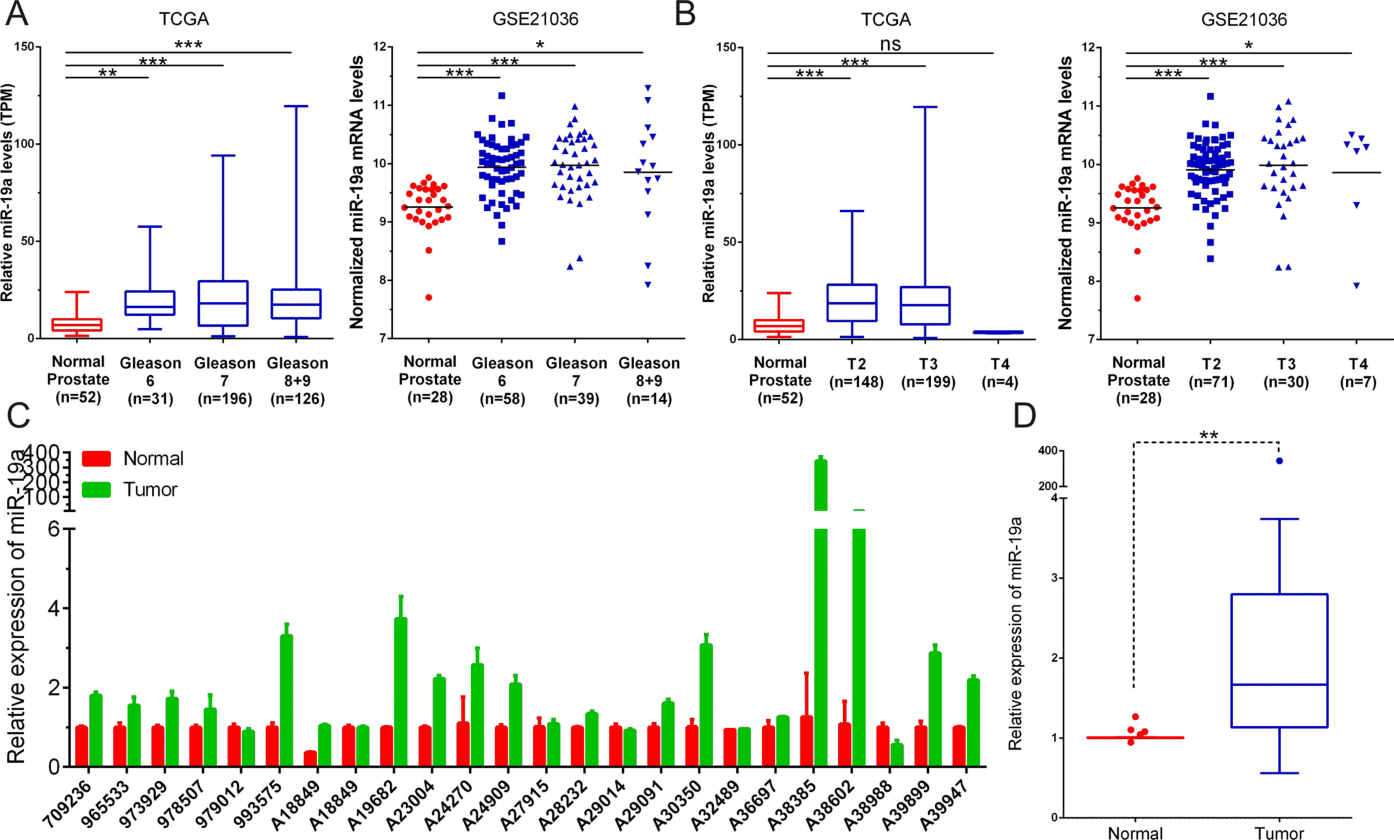
MiR-19a is up-regulated in prostate cancer tissue samples (**A**) MiR-19a expression levels of prostate cancer according to biopsy Gleason scores and in TCGA and GSE21036. (**B**) MiR-19a expression levels of prostate cancer according to preoperative clinical stages in TCGA and GSE21036. (**C**–**D**) Quantitative RT-PCR was performed to evaluate the expression levels of miR-19a from 24 prostate cancers specimens compared with their corresponding adjacent prostate tissues. Expression levels of miR-19a were normalized to that of RNU6.

To elucidate the effects of miR-19a on prostate cancer progression, we analyzed the miR-19a level in TCGA and GSE21036 based on their Gleason score and T staging in detail. Analysis of TCGA database showed that the significantly higher expression of miR-19a was found in Gleason 6 (*P* < 0.01), Gleason 7 (*P* < 0.001) and Gleason 8+9 (*P* < 0.001) patients compared to the matched normal controls (Figure 2A). We then categorized these patients based on T staging and found that tumors of T2 (*P* < 0.001) and T3 (*P* < 0.001) had higher expression of miR-19a compared to the match normal tissues (Figure 2B). We also drew the similar conclusion from GSE21036 database that the significantly higher expression of miR-19a was found in Gleason 6 (*P* < 0.001), Gleason 7 (*P* < 0.001), Gleason 8+9 (*P* < 0.05), T2 (*P* < 0.001), T3 (*P* < 0.001) and T4(*P* <0.05) patients compared to the matched normal controls (Figure 2A–2B).

To further validate this result, we performed qRT-PCR to evaluate miR-19a levels in 24 paired PCa samples we have collected. Consistent with former analysis, miR-19a expression was significantly risen up in PCa tissues when compared with their corresponding adjacent tissues (Figure 2C–2D). These results highlighted the potential role of miR-19a as a novel diagnosis and therapeutic biomarker.

### MiR-19a promotes cell proliferation and migration in PCa cells

**Figure 3:**
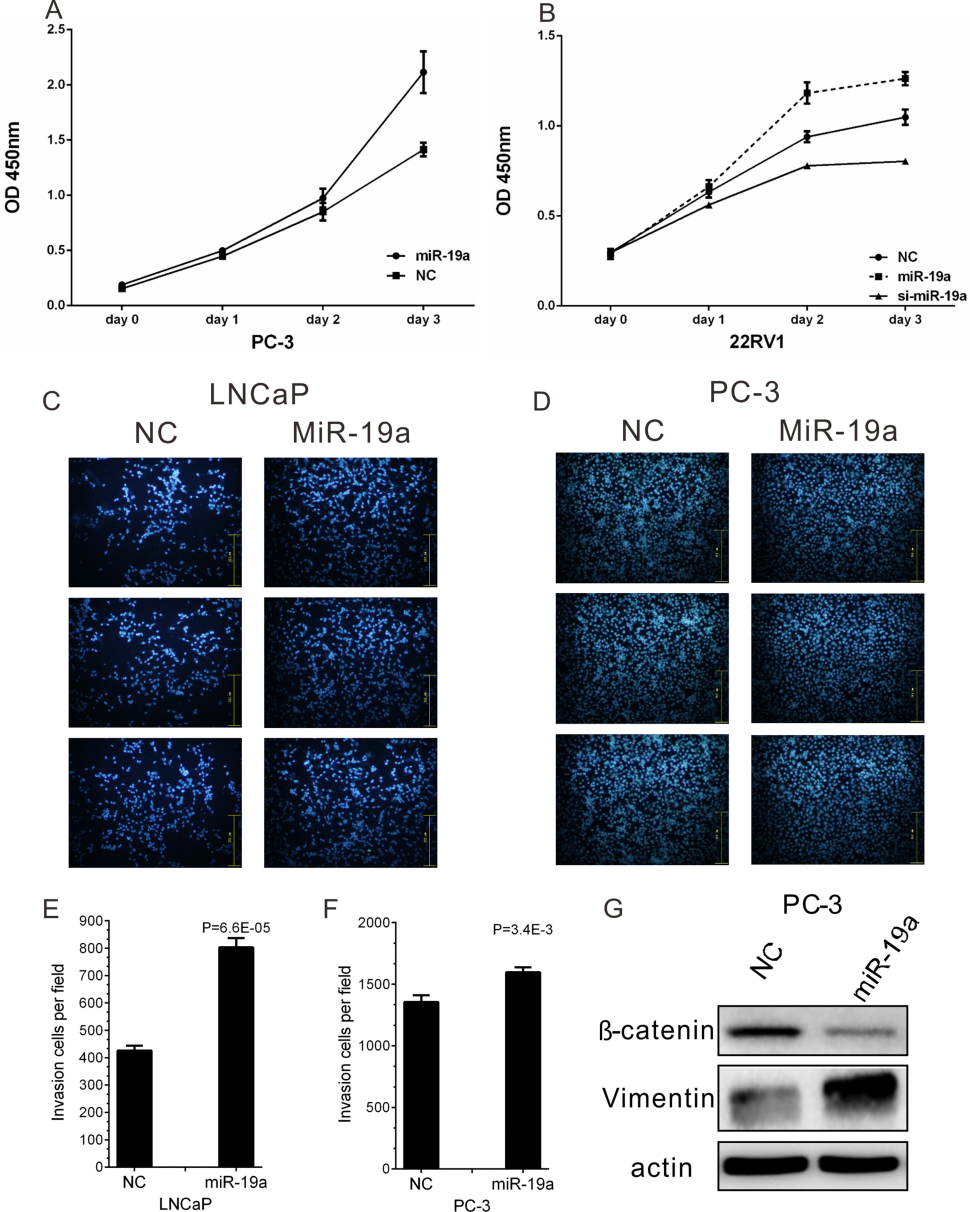
MiR-19a promotes cell proliferation and migration of PCa cells (**A**–**B**) Cell proliferation analysis was performed with CCK-8 assay in PC-3 (A) and 22Rv1 (B) cells. Cells transfected with miR-19a mimics or miR-19a inhibitor were seeded into 96-well plate at 5000 cells/well and examined at different time point. (**C**–**F**) Migration of LNCaP (C) and PC-3 (D) cells after miR-19a and NC transfection was counted by transwell assay. The invasion cell number of LNCaP (E) and PC-3 (F) was presented in histogram. (**G**) Western blot analyses of EMT markers for PC-3 cells after 48 h of miR-19a mimics transfection. Data are presented as the mean ± SD (*n* = 3). Significance was defined as *P* < 0.05 (^*^P < 0.05; ^**^P < 0.01; ^***^P < 0.001); ns means not significant.

To investigate the biological relevance of miR-19a up-regulation in PCa, the effects of miR-19a mimics and inhibitor on cell proliferation were studied using CCK-8 assay in PC-3 and 22Rv1 cells every 24h for 3 days. The expression levels of miR-19a after miR-19a mimics and inhibitor transfection in LNCaP, 22Rv1, DU145 and PC-3 were examined by qRT-PCR (Supplementary Figure 1). The results indicated miR-19a-transfected PC-3 and 22Rv1 cells showed significantly increased rate of cell growth compared with the cell growth of negative control cells, while loss-of-function showed opposite phenotypes of 22Rv1 cells (Figure 3A–3B).

Furthermore, the effect of miR-19a on PCa cell migration was also confirmed in LNCaP and PC-3 cells which were transfected with miR-19a mimics. The migration results showed over-expression of miR-19a significantly increased migration ability of LNCaP and PC-3 cells (Figure 3C–3D), and the invasion cell number per field was remarkedly increased after miR-19a transfection (Figure 3E–3F). Furthermore, western blotting also confirmed overexpression of miR-19a could lead to down-regulation of E-catenin and up-regulation of Vimentin (Figure 3G). The results above showed miR-19a promoted migration in PCa cells

### MiR-19a promotes cell cycle and suppresses apoptosis in PCa cells

**Figure 4:**
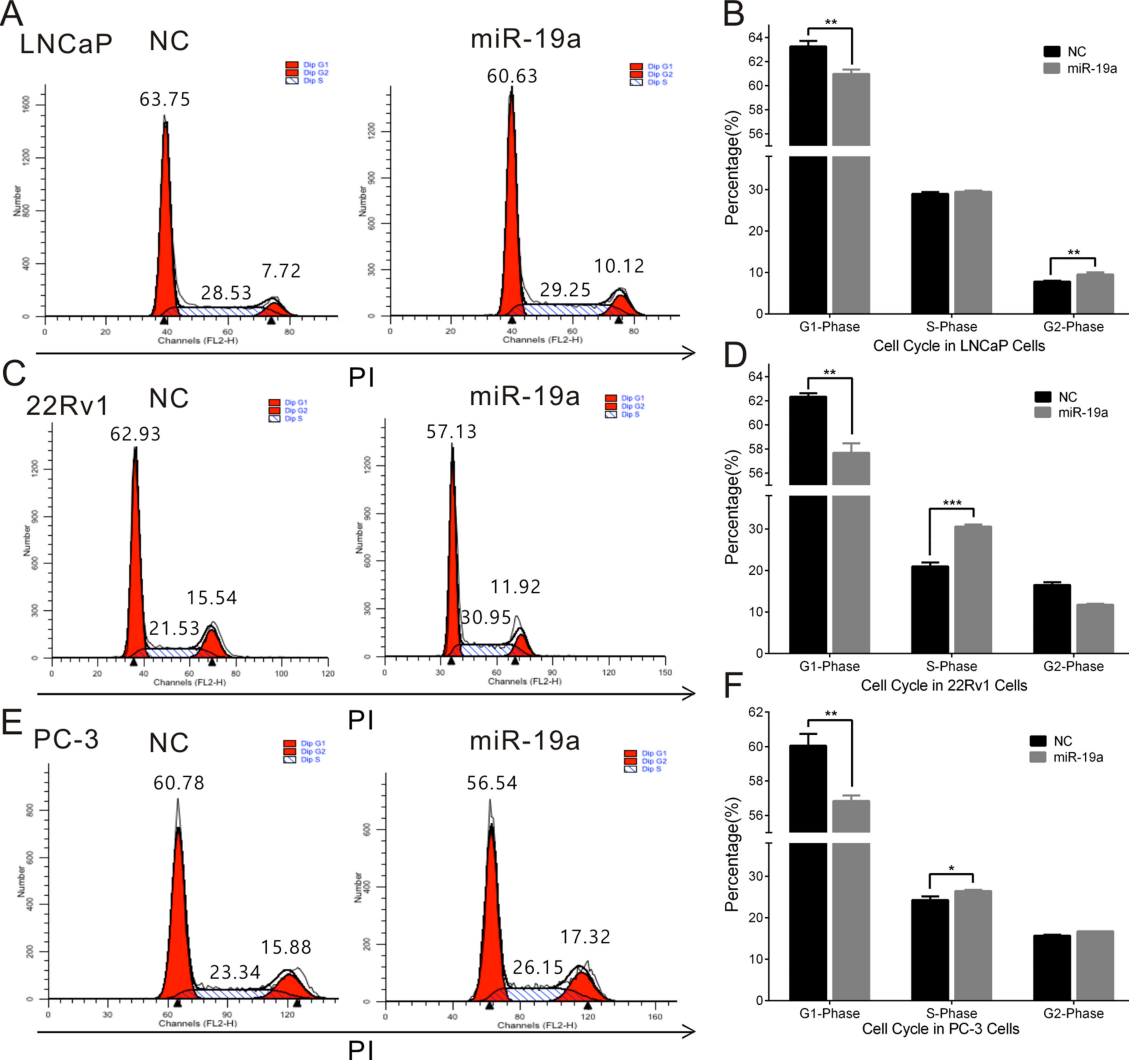
MiR-19a promotes cell cycle in PCa cells (**A**–**F**) Cell cycle assay was performed in LNCaP (A, B), 22Rv1 (C, D) and PC-3 (E, F) cells. Cells were transfected with miR-19a for 48h, stained with PI and evaluated with a FACScalibur flow cytometer. Data are presented as the mean ± SD (*n* = 3). Significance was defined as *P* < 0.05 (^*^P < 0.05; ^**^P < 0.01; ^***^P < 0.001); ns means not significant.

To further explore the cause of the promotion in cell proliferation, miR-19a-transfected LNCaP, 22Rv1 and PC-3 cells were stained by PI, and cell cycle was analyzed by flow cytometry. We observed miR-19a could induce the decreases in G1 phase of LNCaP, 22Rv1 and PC-3 cells, the increases in S phase of 22Rv1, PC-3 and G2 phase of LNCaP (Figure 4A–4F).

**Figure 5:**
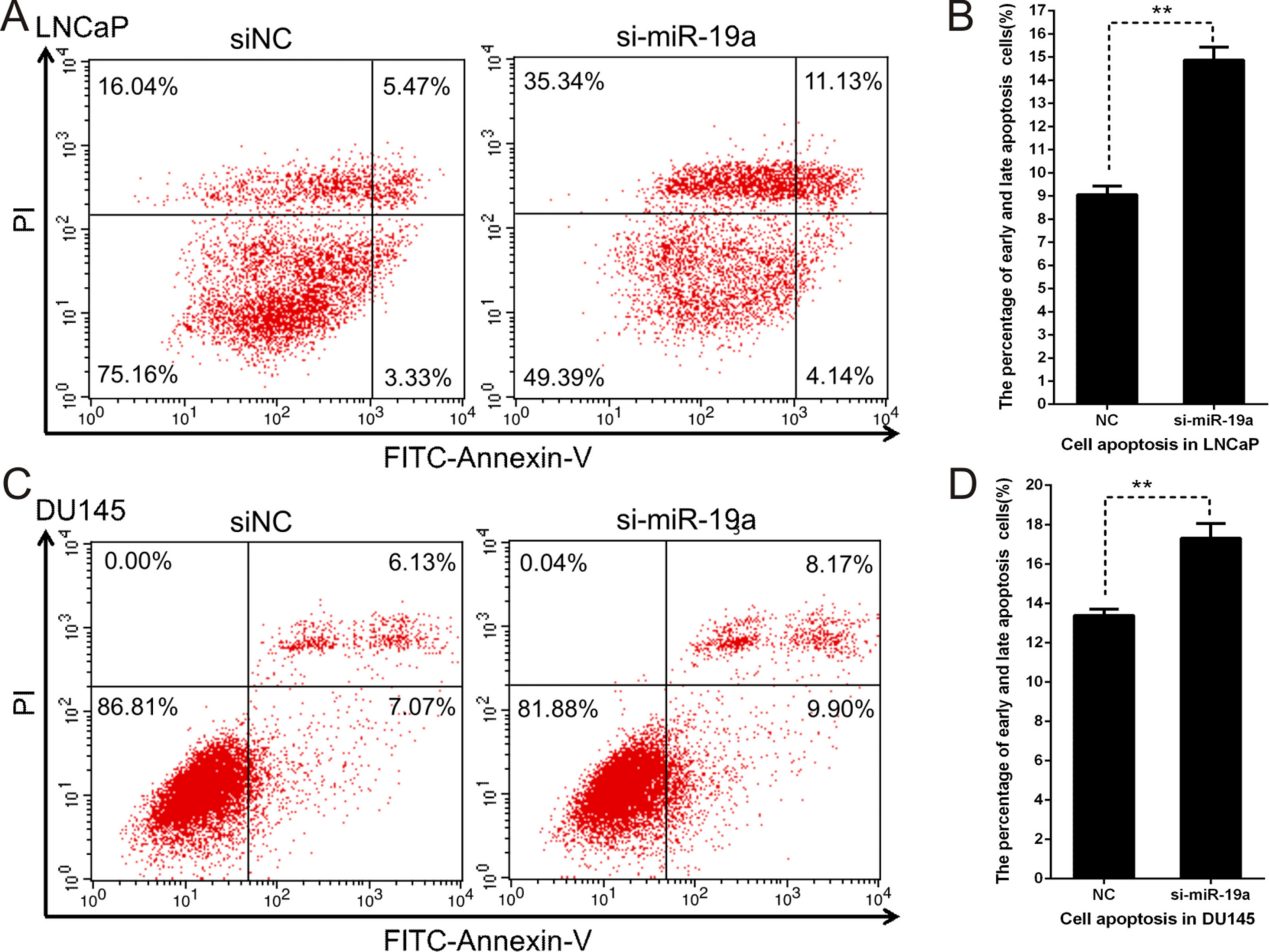
Downregulation of miR-19a induces apoptosis in PCa cells (**A**–**D**) Cell apoptosis assay was performed with flow cytometer. Cells were transfected with miR-19a inhibitor for 48 h, and subjected to cell apoptosis assay. Down-regulation of miR-19a in LNCaP (A, B) and DU145 (C, D) cells increased the fraction of both early apoptotic cells and late apoptotic cells. In A and C, the lower left region represented normal cells, lower right region represented early apoptosis cells, upper right region represented late apoptosis cells, and upper left region represented necrosis cells. Data are presented as the mean ± SD (*n* = 3). Significance was defined as *P* < 0.05 (^*^P < 0.05; ^**^P < 0.01; ^***^P < 0.001); ns means not significant.

To investigate the apoptotic effects of miR-19a in PCa, LNCaP and DU145 cells transfected with miR-19a inhibitor were stained by Annexin V and PI, and cell apoptosis was analyzed by flow cytometry. Indeed, compared with the inhibitor control, the early and late apoptotic rates were higher in LNCaP and DU145 cells transfected with miR-19a inhibitor (Figure 5A–5D).

### VPS37A is a direct target mRNA of miR-19a in PCa cells

**Figure 6:**
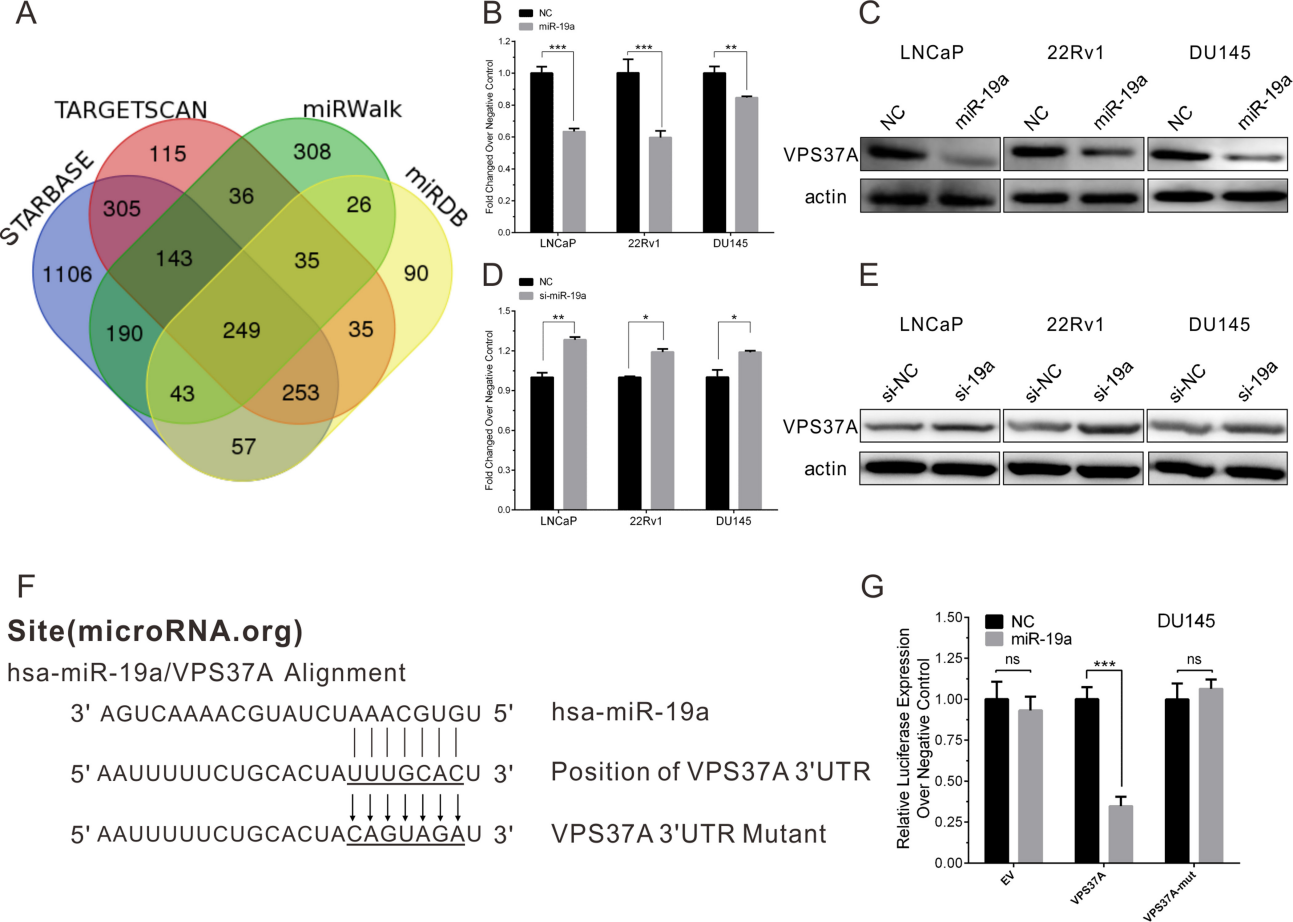
MiR-19a directly targets VPS37A in PCa cells (**A**) Venn diagrams for miR-19a targeted genes using four open-access algorithms, including TargetScan, miRanda, miRwalk, and starBase. (**B**–**C**) qRT-PCR and western blotting analysis of VPS37A after miR-19a mimics transfection in LNCaP, 22Rv1 and DU145 as indicated. β-actin was used as internal control. (**D**–**E**) qRT-PCR and western blotting analysis of VPS37A after miR-19a inhibitor transfection in LNCaP, 22Rv1 and DU145 as indicated. β-actin was used as internal control. (**F**) Sequence alignment of human miR-19a and 3′-UTR of VPS37A using microRNA.org. The seed sequence of miR-19a (top) matches 3′-UTR of VPS37A (middle). Bottom is the mutation of the 3′-UTR of VPS37A used in luciferase reporter construct. (**G**) Luciferase assay in DU145 cells. MiR-19a over-expression decreased the luciferase activity of VPS37A, but did not affect that of EV and VPS37A-mut. Data are presented as the mean ± SD (*n* = 3). Significance was defined as *P* < 0.05 (^*^P < 0.05; ^**^P < 0.01; ^***^P < 0.001); ns means not significant.

To gain further insight into molecular mechanisms and pathways regulated by oncogenic miR-19a in PCa, we used four computational algorithms (including starBase [21], TargetScan [22], miRWalk [23] and miRDB [24]) to search for the potential targets of miR-19a. A total of 249 potential target mRNAs were identified (Figure 6A). Among 249 potential target mRNAs, we picked VPS37A out as the target of miR-19a according to the possibility and their biological function. To evaluate the impact of the miR-19a in the expression of VPS37A, we performed qRT–PCR and western blotting, which showed overexpression of miR-19a significantly inhibited the mRNA and protein expression level of VPS37A in LNCaP, 22Rv1 and DU145 cells (Figure 6B–6C). Similarly, the mRNA and protein levels of VPS37A were significantly up-regulated in LNCaP, 22Rv1 and DU145 cells transfected by miR-19a inhibitor (Figure 6D–6E).

To confirm whether VPS37A is a direct target gene of miR-19a, the miR-19a putative binding sites from wild-type 3′-UTR of VPS37A were cloned into downstream of the *Renilla* luciferase reporter gene in the psiCHECK2 vector (Figure 6F). DU145 cells were co-transfected with psiCHECK2-VPS37A and miR-19a mimics, while empty and mutant vector were served as controls. By measuring the luciferase activity, miR-19a showed significant reduction in luciferase expression compared to miR-NC (Figure 6G). As for empty and mutant vector of psiCHECK2, there were no statistical significance between miR-19a and miR-NC. Taking together, we could draw the conclusion that miR-19a binds to 3′-UTR of VPS37A to down-regulate the expression of VPS37A.

### VPS37A attenuates the effect of miR-19a as an oncogene

**Figure 7:**
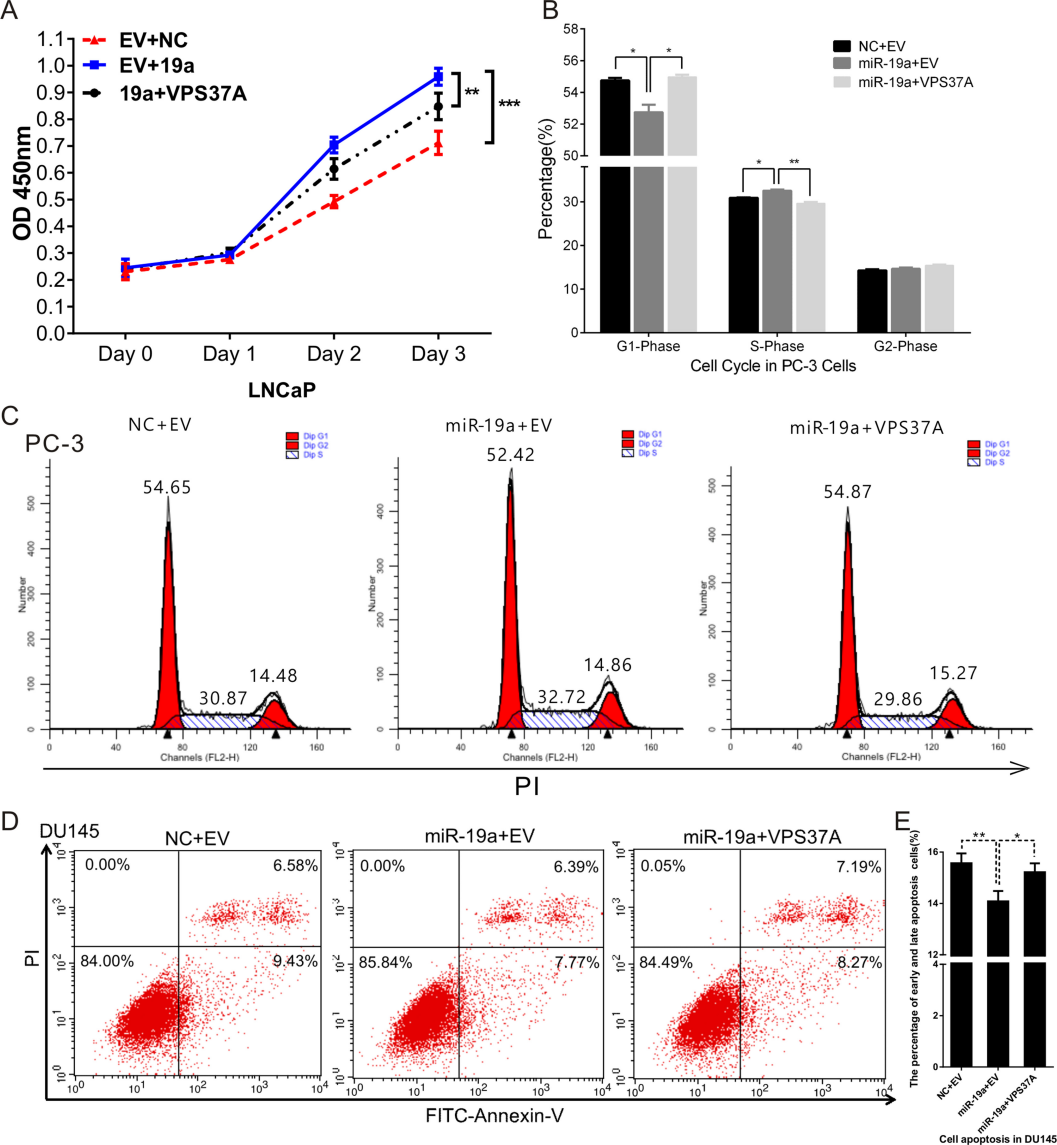
MiR-19a regulates proliferation, cell cycle and apoptosis through VPS37A (**A**) Cell proliferation analysis was performed with CCK-8 assay in LNCaP co-transfected with miR-19a and pcDNA3.1(+)-VPS37A. (**B**–**C**) Cell cycle assay was performed in PC-3 cells. Cells were co-transfected with miR-19a and pcDNA3.1(+)-VPS37A for 48h. (**D**–**E**) Cell apoptosis assay was performed in DU145 cells co-transfected with miR-19a and pcDNA3.1(+)-VPS37A for 48 h with flow cytometer. Data are presented as the mean ± SD (*n* = 3). Significance was defined as P < 0.05 (^*^P < 0.05; ^**^P < 0.01; ^***^P < 0.001); ns means not significant.

To investigate whether miR-19a plays its oncogenic role of PCa cells by negatively regulating VPS37A, we constructed a VPS37A overexpression pcDNA3.1(+) vector and performed rescue experiments in PCa cells in proliferation, cell cycle and apoptosis. PCa cells were co-transfected with miR-19a mimics and VPS37A-pcDNA3.1(+) vector, and then assays were conducted. CCK-8 results demonstrated VPS37A inhibited the effect of miR-19a in enhancing proliferation in DU145 cells (Figure 7A). Similarly, flow cytometry assays also showed VPS37A overexpression could significantly up-regulate G1 phase and down-regulated S phase in PC-3 cells compared to miR-19a overexpressing cells (Figure 7B–7C). In addition, the apoptosis results indicated that VPS37A significantly induced apoptosis of DU145 cells compare to miR-19a-over-expressing cells (Figure 7D–7E). These data suggest that miR-19a is related to the regulation of cell proliferation, cell cycle and apoptosis at least partially through VPS37A.

### MiR-19a is inversely correlated with the level of VPS37A in PCa specimens

**Figure 8:**
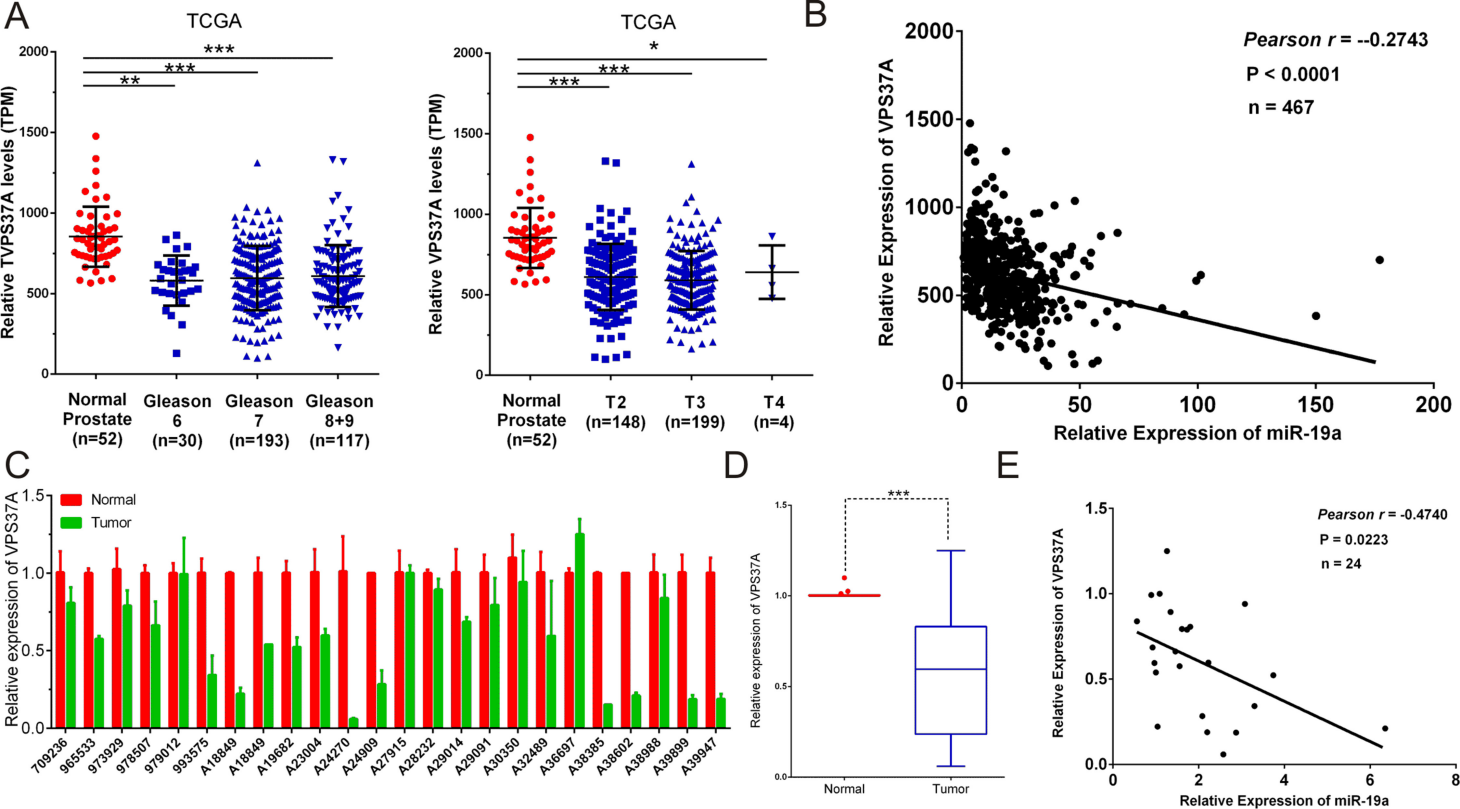
Correlations between the expression levels of miR-19a and VPS37A (**A**) MiR-19a expression levels of prostate cancer according to biopsy Gleason scores and preoperative clinical stages in TCGA. (**B**) Correlation plots of miR-19a and VPS37A by Pearson's product-moment correlation coefficient in TCGA. (**C**–**D**) Quantitative RT-PCR was performed to evaluate the expression levels of VPS37A from 24 prostate cancers specimens compared with their corresponding adjacent prostate tissues. β-actin was used as internal control. (**E**) Correlation plots of miR-19a and VPS37A by Pearson's product-moment correlation coefficient in PCa specimens. Data are presented as the mean ± SD (*n* = 3). Significance was defined as *P* < 0.05 (^*^P < 0.05; ^**^P < 0.01; ^***^P < 0.001); ns means not significant.

Given that VPS37A was a downstream target of miR-19a, we further investigated whether the expression level of miR-19a was inversely correlated with the mRNA level of VPS37A in PCa specimens. We first analyzed the mRNA level of VPS37A in TCGA database. Not surprisingly, the significantly higher expression of VPS37A was found in Gleason 6 (*P* < 0.01), Gleason 7 (*P* < 0.001), Gleason 8+9 (*P* < 0.001), T2 (*P* < 0.001), T3 (*P* < 0.001) and T4 (*P* < 0.05) patients compared to the corresponding normal controls (Figure 8A).

The Pearson's rank correlation coefficient analyses on TCGA data revealed the inverse correlation of expression of miR-19a and VPS37A (*Pearson r* = -0.2743, *P*<0.0001; Figure 8B). To further validate it, we performed qRT-PCR on VPS37A mRNA levels of PCa specimens that we have collected and it came out with the same results which indicated VPS37A level was downregulated in PCa tissues (Figure 8C–8D) and the Pearson's rank correlation coefficient analyses showed an inverse correlation between miR-19a and VPS37A expression (*Pearson r* = -0.4740, *P* = 0.0223; Figure 8E).

### Upregulation of miR-19a and downregulation of VPS37A correlate with poor survival in PCa

**Figure 9:**
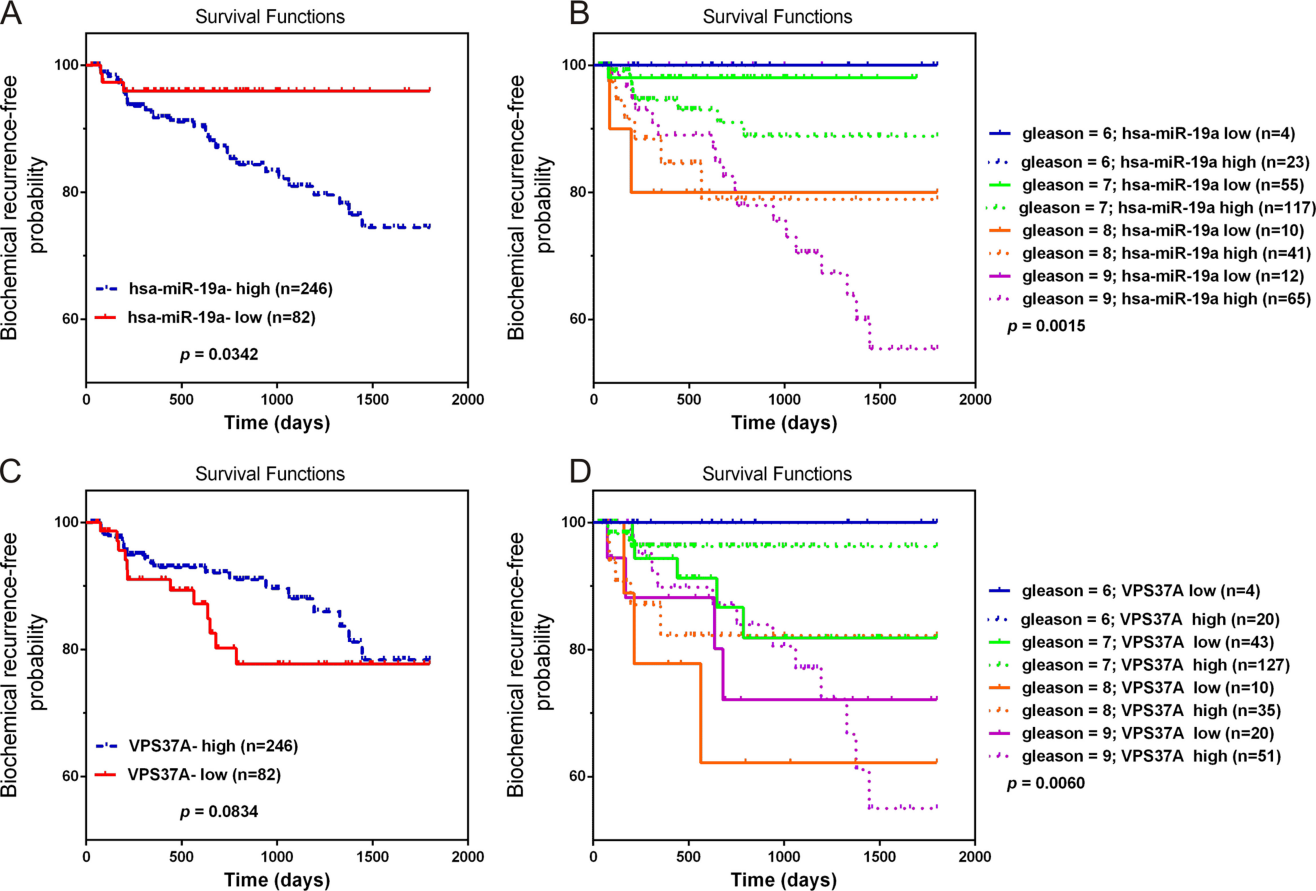
Overexpression of miR-19a and downregulation of VPS37A predict poor prognosis in PCa (**A**–**B**) Kaplan-Meier curves for survival time after radical prostatectomy in patients with prostate cancer according to expression of over-all miR-19a (A) and their pathological stage (B). (**C**–**D**) Kaplan-Meier curves for survival time after radical prostatectomy in patients with prostate cancer according to expression of over-all VPS37A (C) and their pathological stage (D).

To determine the prognostic value of miR-19a and VPS37A, Kaplan-Meier survival curve analyses on TCGA database were performed. The results revealed miR-19a-high phenotypes were associated with poor prognosis with biochemical recurrence-free probability (*P* = 0.0342; Figure 9A). As for VPS37A, although the *P*-value was not significant, there was still a trend that downregulation of VPS37A was associated with poor outcome (*P* = 0.0834; Figure 9C). We also combined miR-19a and VPS37A expression with pathological stage to stratify the possibility of biochemical recurrence. The miR-19a-high group had a significantly increased possibility of biochemical recurrence among the subsets of Gleason Scores 7 and 9 tumors compared with those with miR-19a-low expression (log rank *P* = 0.0111, Gleason Scores 7; log rank *P* = 0.0007, Gleason Scores 9; Figure 9B). The VPS37A-low group had a significantly increased possibility of biochemical recurrence among the subsets of Gleason Scores 7 and 8 tumors compared with those with VPS37A-high expression (log rank *P* = 0.0111, Gleason Scores 7; log rank *P* = 0.0007, Gleason Scores 8; Figure 9D). These results highlighted the role of miR-19a and VPS37A as significant prognostic markers.

## DISCUSSION

Prostate cancer (PCa) is the second most common cancer in male worldwide [1]. However, the precise mechanism of PCa progression still remains largely unclear. To investigate the miRNAs potentially involved in PCa progression, we profiled miRNAs expressing aberrantly in PCa from TCGA, GSE21036 and GSE76260 and found out 16 significantly up-regulated miRNAs and 13 significantly down-regulated miRNAs compared with normal prostate. Of note, our results were consistent with previous reports. For example, miR-32 and miR-183 had been reported to be up-regulated in PCa. Interestingly, some miRNAs were reported to have opposite effect. For example, miR-200c, one of the most up-regulated microRNAs in PCa according to our study, inhibited migration, invasion and EMT in PCa cells [3, 4]. Our studies provided a general understanding of miRNAs participating PCa progression. Furthermore, we focused on miR-19a which was one of the most upregulated in PCa.

MiR-19a, a member of miR-17–92 cluster, is located at 13q31. Previously, miR-19a was found to be up-regulated in several cancers, such as colorectal cancer [25], lung cancer [26], breast cancer [27] and multiple myeloma [28]. MiR-19a is considered to be an oncogene in several solid cancers. For example, miR-19a was reported to play oncogenic role in gastric cancer by targeting the tumor suppressor MXD1 [29] ,in cervical carcinoma by targeting CUL5 [30] and in pancreatic cancer by downregulating RHOB [31] promote and. In PCa, several groups had also indicated the potential roles of miR-19a in tumor progression. For example, Ottman et al. reported miR-17-92a cluster exhibited tumor suppressor effects in PCa [32]. Lu et al. found out miR-19a regulated proliferation and apoptosis by targeting BTG1 only in castration-resistant PCa cells [33]. Of note, we and some other groups had showed that miR-19a was up-regulated in PCa [34–36]. However, until recently, the mechanism underlying miR-19a regulation in PCa had not been fully elucidated. Our study showed that miR-19a was highly expressed in PCa specimens compared with normal prostate tissues. In present study, we found out overexpression of miR-19a could exerted its oncogenic effects by promoting cell proliferation, cell cycle, cell growth and cell migration. To our knowledge, it's the first time to clarify the role of miR-19a in both androgen-responsive and castration-resistant PCa cell lines.

In this study, we identified VPS37A as a direct target of miR-19a. MiR-19a directly combined to 3′-UTR of VPS37A to downregulate VPS37A in both mRNA and protein level. VPS37A, also named as HCRP1 (hepatocellular carcinoma related protein 1), belongs to the VPS37 family [37]. It has been reported that VPS37A was downregulated and could be an independent predictor in hepatocellular carcinoma (HCC) [38], non-small cell lung cancer [39] and oropharyngeal cancer [40]. In HCC, downregulation of VPS37A promoted hepatocellular carcinoma cell migration and invasion by induction of EGFR activation and epithelial-mesenchymal transition [41, 42]. In breast cancer, VPS37A was downregulated and inhibited breast cancer metastasis through downregulating EGFR phosphorylation [43]. In this study, we found VPS37A overexpression could significantly inhibit cell proliferation and promote cell apoptosis in PCa cells. Of note, the rescue experiments in cell proliferation, cell cycle and apoptosis also indicated miR-19a played its role via VPS37A. For the first time, these results demonstrated miR-19a could target VPS37A to promote PCa progression.

Furthermore, the clinical values of miR-19a and VPS37A were revealed by our study. Public data analysis showed miR-19a was up-regulated and VPS37A was down-regulated in PCa samples. Moreover, up-expression of miR-19a and low-expression of VPS37A were observed in our 24 PCa specimen. Moreover, miR-19a-high and VPS37A-low phenotypes were associated with poor prognosis with biochemical recurrence-free probability. In summary, miR-19a and VPS37A might be potential markers for PCa.

Collectively, the main aim of this research is to explore the effect of miR-19a on PCa cells and find out the potential mechanisms of it. Notably, we identified VPS37A as the direct target of miR-19a. In conclusion, miR-19a could accelerate tumorigenesis by inhibiting VPS37A and act as a potential diagnostic and therapeutic target for PCa. These findings may help us understand the important role of miR-19a in promoting PCa progression and provide a new diagnostic and therapeutic biomarker for castration-resistant PCa.

## MATERIALS AND METHODS

### Cell culture

LNCaP cells were purchased from the American Type Cult. Collection (Manassas, USA) which were confirmed by short tandem repeat (STR) analysis. 22RV1, DU145 and PC-3 were obtained from Cell Bank of Chinese Academy of Sciences (Shanghai, China) where they were authenticated by mycoplasma detection, DNA-Fingerprinting, isozyme detection and cell vitality detection. All experiments were carried out with cell lines at passages below 30. The four prostate cancer cell lines were maintained in RPMI 1640 medium (Corning, USA) supplemented with 10% FBS (Hyclone, USA) and cultured at 37°C in 5% CO_2_.

### RNA interference and transient transfection

Synthetic miR-19a mimic (miR-19a) and its scrambled control miRNA (miR-NC) were purchased from GenePharma (Shanghai, China), and used at the concentration of 50nM. Synthetic miR-19a inhibitor (si-19a) and its scrambled control miRNA (inhibitor NC, siNC) were purchased from GenePharma (Shanghai, China), and used at the concentration of 50nM. Transfection was carried out with Lipofectamine 2000 Transfection Reagent (Life, USA) according to the manufacturer's procedures. The Opti-MEM medium and Lipofectamine 2000 were both purchased from Life Technologies.

### RNA isolation and real-time qPCR

Total RNA was extracted from PCa cells with Trizol (Invitrogen, CA, USA), and reverse transcription was performed according to the manual of NovoScript^®^ 1st Strand cDNA Synthesis SuperMix (Novoprotein Scientific Inc. China). Real-time quantitative PCR was carried out with AceQ qPCR SYBR Green Master Mix (Vazyme Biotech co., ltd) on LightCycler 480II (Roche, Basel, Switzerland) instrument. Specific primers for mature miR-19a were from GenePharma (Shanghai, China). Primers used for qRT-PCR were listed in Supplementary Table 1 (Supplementary Table 3). The Ct values were normalized using β-actin or RNU6 as internal control to estimate the different expression of genes. Relative mRNA expression was calculated using the 2^-ΔΔCt^ method. Each sample was run in triplicate to ensure quantitative accuracy.

### Western blotting analysis

Western blot was performed as described previously [44] with antibodies against VPS37A (1:1000, Proteintech, Chicago, USA), and β-actin (1:4000, Sigma-Aldrich). Goat anti-mouse IgG-HRP and goat anti-rabbit IgG-HRP (Sigma-Aldrich, USA) secondary antibodies were used to visualize bands using Amersham ECL Prime (GE Healthcare, UK). Signal intensity of Western blots was quantified by Quantity One Software (Bio-Rad, USA).

### Reporter constructs and luciferase assay

700-bp nucleotide sequences corresponding to portion of the 3′-UTR of VPS37A, including the conserved predicted binding site (seed sequence) for miR-19a, were inserted into psi-CHECK2, Dual-Luciferase miRNA Target Expression Vector (Promega, USA) within the XhoI/ NotI sites. Mutagenesis was performed using Mut Express® II Fast Mutagenesis Kit V2 (Vazyme, USA). All insertions were verified by sequencing. The relative luciferase activity was measured by Dual-Luciferase Reporter Assay System (Promega, USA) 24h after transfection.

### Cell proliferation assay

Cell proliferation was assessed by Cell Counting Kit-8 (CCK-8, Dojindo Laboratories, Japan) in octuplicate according to the manufacturer's instructions. Absorbance was measured at 450 nm with Microplate Reader ELx808 (Bio-Tek, USA). The absorbance at 615 nm was used as a reference.

### Cell cycle and apoptosis assay

Cells were harvested 48 h after transfection. For cycle assay, cells were incubated with 0.03% triton X-100 and propidium iodide (PI) (50 ng/mL) for 15 min; the percentages of cells in different phases of cell cycle were measured with a FACScalibur flow cytometer (BD, USA) and analyzed with ModFit software (Verity Software House, USA). For apoptosis assay, cells were assayed with FITC Annexin V Apoptosis Detection Kit (BD, USA) and analyzed by flow cytometry.

### Cell migration assay

Cells (2 × 10^4^/well) after transfection were seeded with media containing 1% FBS into the upper chamber of transwell filter (8-μm pore size, 6.5-mm diameter; Corning, MA, USA) on a 24-well plate. Medium containing 10% FBS was used as attractant and added to the lower well of the plate. Plates were incubated in a humidified incubator at 37°C and 5% CO_2_ for 120 h. Non-migrating cells on the upper side of the filter were removed using cotton swabs, and cells that migrated were fixed with methanol, stained with Giemsa stain, and counted under a microscope. Cells in five randomly chosen fields per trans-well filter were counted to quantify the average number of migration. Each experiment was repeated three times.

### Tissue collection

The trial was approved by the Research Ethics Committee of Fudan University Shanghai Cancer Center and verbal consent was obtained from all patients. Twenty-four prostatic adenocarcinoma tissue samples and matched normal prostate tissues were used for an extra evaluation by qRT-PCR. All samples were collected from Fudan University Shanghai Cancer Center, between January 2010 and December 2016. The prostate cancer patients from whom the tissues were obtained underwent radical prostatectomy and did not receive any pre-operation treatment. The histopathological features of tumor specimens were classified according to the Gleason score system and 2002 TNM classification system.

### Statistical analysis

The numerical data were presented as mean ± standard deviation (SD) of at least three determinations. Statistical comparisons between groups of normalized data were performed using *T*-test or Mann–Whitney *U*-test according to the test condition. A *p* < 0.05 was considered statistical significance with a 95% confidence level.

## SUPPLEMENTARY MATERIALS FIGURE AND TABLES



## Abbreviations

3′-UTR: 3′-untranslated region
FBS: Fetal bovine serum
HCC: Hepatocellular carcinoma
HCRP1: Hepatocellular carcinoma related protein 1
miRNA: microRNA
Mut: Mutant
NC: Negative control
PCa: Prostate cancer
PSA: Prostate-specific antigen
qRT-PCR: Quantitative real time polymerase chain reaction
VPS37A: Vacuolar protein sorting 37A.

## References

[1] Torre LA, Bray F, Siegel RL, Ferlay J, Lortet-Tieulent J, Jemal A (2015). Global cancer statistics, 2012. CA Cancer J Clin.

[2] Siegel RL, Miller KD, Jemal A (2015). Cancer statistics, 2015. CA Cancer J Clin.

[3] Sartor AO, Hricak H, Wheeler TM, Coleman J, Penson DF, Carroll PR, Rubin MA, Scardino PT (2008). Evaluating localized prostate cancer and identifying candidates for focal therapy. Urology.

[4] Lu-Yao GL, Albertsen PC, Moore DF, Shih W, Lin Y, DiPaola RS, Barry MJ, Zietman A, O’Leary M, Walker-Corkery E, Yao SL (2009). Outcomes of localized prostate cancer following conservative management. JAMA.

[5] D’Amico AV, Chen MH, Roehl KA, Catalona WJ (2004). Preoperative PSA velocity and the risk of death from prostate cancer after radical prostatectomy. N Engl J Med.

[6] Macoska JA, Begley LA, Dunn RL, Siddiqui J, Wei JT, Sarma AV (2008). Pilot and feasibility study of serum chemokines as markers to distinguish prostatic disease in men with low total serum PSA. Prostate.

[7] Ambros V (2004). The functions of animal microRNAs. Nature.

[8] Bartel DP (2004). MicroRNAs: genomics, biogenesis, mechanism, and function. Cell.

[9] Lagos-Quintana M, Rauhut R, Lendeckel W, Tuschl T (2001). Identification of novel genes coding for small expressed RNAs. Science.

[10] Huang Y, Shen XJ, Zou Q, Wang SP, Tang SM, Zhang GZ (2011). Biological functions of microRNAs: a review. J Physiol Biochem.

[11] Wu W, Sun M, Zou GM, Chen J (2007). MicroRNA and cancer: Current status and prospective. Int J Cancer.

[12] Sotillo E, Thomas-Tikhonenko A (2011). Shielding the messenger (RNA): microRNA-based anticancer therapies. Pharmacol Ther.

[13] Catto JW, Alcaraz A, Bjartell AS, De Vere White R, Evans CP, Fussel S, Hamdy FC, Kallioniemi O, Mengual L, Schlomm T, Visakorpi T (2011). MicroRNA in prostate, bladder, and kidney cancer: a systematic review. Eur Urol.

[14] Andorfer CA, Necela BM, Thompson EA, Perez EA (2011). MicroRNA signatures: clinical biomarkers for the diagnosis and treatment of breast cancer. Trends Mol Med.

[15] Kota J, Chivukula RR, O’Donnell KA, Wentzel EA, Montgomery CL, Hwang HW, Chang TC, Vivekanandan P, Torbenson M, Clark KR, Mendell JR, Mendell JT (2009). Therapeutic microRNA delivery suppresses tumorigenesis in a murine liver cancer model. Cell.

[16] Wan X, Huang W, Yang S, Zhang Y, Zhang P, Kong Z, Li T, Wu H, Jing F, Li Y (2016). Androgen-induced miR-27A acted as a tumor suppressor by targeting MAP2K4 and mediated prostate cancer progression. Int J Biochem Cell Biol.

[17] Wan X, Pu H, Huang W, Yang S, Zhang Y, Kong Z, Yang Z, Zhao P, Li A, Li T, Li Y (2016). Androgen-induced miR-135a acts as a tumor suppressor through downregulating RBAK and MMP11, and mediates resistance to androgen deprivation therapy. Oncotarget.

[18] Guan H, Liu C, Fang F, Huang Y, Tao T, Ling Z, You Z, Han X, Chen S, Xu B, Chen M (2017). MicroRNA-744 promotes prostate cancer progression through aberrantly activating Wnt/beta-catenin signaling. Oncotarget.

[19] Colaprico A, Silva TC, Olsen C, Garofano L, Cava C, Garolini D, Sabedot TS, Malta TM, Pagnotta SM, Castiglioni I, Ceccarelli M, Bontempi G, Noushmehr H (2016). TCGAbiolinks: an R/Bioconductor package for integrative analysis of TCGA data. Nucleic Acids ReS.

[20] Taylor BS, Schultz N, Hieronymus H, Gopalan A, Xiao Y, Carver BS, Arora VK, Kaushik P, Cerami E, Reva B, Antipin Y, Mitsiades N, Landers T (2010). Integrative genomic profiling of human prostate cancer. Cancer Cell.

[21] Yang JH, Li JH, Shao P, Zhou H, Chen YQ, Qu LH (2011). starBase: a database for exploring microRNA-mRNA interaction maps from Argonaute CLIP-Seq and Degradome-Seq data. Nucleic Acids Res.

[22] Lewis BP, Burge CB, Bartel DP (2005). Conserved seed pairing, often flanked by adenosines, indicates that thousands of human genes are microRNA targets. Cell.

[23] Dweep H, Sticht C, Pandey P, Gretz N (2011). miRWalk--database: prediction of possible miRNA binding sites by “walking” the genes of three genomes. J Biomed Inform.

[24] Wong N, Wang X (2015). miRDB: an online resource for microRNA target prediction and functional annotations. Nucleic Acids ReS.

[25] Liu Y, Liu R, Yang F, Cheng R, Chen X, Cui S, Gu Y, Sun W, You C, Liu Z, Sun F, Wang Y, Fu Z (2017). miR-19a promotes colorectal cancer proliferation and migration by targeting TIA1. Mol Cancer.

[26] Lin Q, Chen T, Lin Q, Lin G, Lin J, Chen G, Guo L (2013). Serum miR-19a expression correlates with worse prognosis of patients with non-small cell lung cancer. J Surg Oncol.

[27] Anfossi S, Giordano A, Gao H, Cohen EN, Tin S, Wu Q, Garza RJ, Debeb BG, Alvarez RH, Valero V, Hortobagyi GN, Calin GA, Ueno NT (2014). High serum miR-19a levels are associated with inflammatory breast cancer and are predictive of favorable clinical outcome in patients with metastatic HER2+ inflammatory breast cancer. PLoS One.

[28] Hao M, Zang M, Wendlandt E, Xu Y, An G, Gong D, Li F, Qi F, Zhang Y, Yang Y, Zhan F, Qiu L (2015). Low serum miR-19a expression as a novel poor prognostic indicator in multiple myeloma. Int J Cancer.

[29] Wu Q, Yang Z, An Y, Hu H, Yin J, Zhang P, Nie Y, Wu K, Shi Y, Fan D (2014). MiR-19a/b modulate the metastasis of gastric cancer cells by targeting the tumour suppressor MXD1. Cell Death DiS.

[30] Xu XM, Wang XB, Chen MM, Liu T, Li YX, Jia WH, Liu M, Li X, Tang H (2012). MicroRNA-19a and -19b regulate cervical carcinoma cell proliferation and invasion by targeting CUL5. Cancer Lett.

[31] Tan Y, Yin H, Zhang H, Fang J, Zheng W, Li D, Li Y, Cao W, Sun C, Liang Y, Zeng J, Zou H, Fu W, Yang X (2015). Sp1-driven up-regulation of miR-19a decreases RHOB and promotes pancreatic cancer. Oncotarget.

[32] Ottman R, Levy J, Grizzle WE, Chakrabarti R (2016). The other face of miR-17-92a cluster, exhibiting tumor suppressor effects in prostate cancer. Oncotarget.

[33] Lu K, Liu C, Tao T, Zhang X, Zhang L, Sun C, Wang Y, Chen S, Xu B, Chen M (2015). MicroRNA-19a regulates proliferation and apoptosis of castration-resistant prostate cancer cells by targeting BTG1. FEBS Lett.

[34] Mo W, Zhang J, Li X, Meng D, Gao Y, Yang S, Wan X, Zhou C, Guo F, Huang Y, Amente S, Avvedimento EV, Xie Y (2013). Identification of novel AR-targeted microRNAs mediating androgen signalling through critical pathways to regulate cell viability in prostate cancer. Plos One.

[35] Wang SY, Shiboski S, Belair CD, Cooperberg MR, Simko JP, Stoppler H, Cowan J, Carroll PR, Blelloch R (2014). miR-19, miR-345, miR-519c-5p serum levels predict adverse pathology in prostate cancer patients eligible for active surveillance. Plos One.

[36] Stuopelyte K, Daniunaite K, Jankevicius F, Jarmalaite S (2016). Detection of miRNAs in urine of prostate cancer patients. Medicina (Kaunas).

[37] Bache KG, Slagsvold T, Cabezas A, Rosendal KR, Raiborg C, Stenmark H (2004). The growth-regulatory protein HCRP1/hVps37A is a subunit of mammalian ESCRT-I and mediates receptor down-regulation. Mol Biol Cell.

[38] Xu Z, Liang L, Wang H, Li T, Zhao M (2003). HCRP1, a novel gene that is downregulated in hepatocellular carcinoma, encodes a growth-inhibitory protein. Biochem Biophys Res Commun.

[39] Du Y, Wang P, Sun H, Yang J, Lang X, Wang Z, Zang S, Chen L, Ma J, Sun D (2016). HCRP1 is downregulated in non-small cell lung cancer and regulates proliferation, invasion, and drug resistance. Tumour Biol.

[40] Perisanidis C, Savarese-Brenner B, Wurger T, Wrba F, Huynh A, Schopper C, Kornek G, Selzer E, Ewers R, Psyrri A, Krainer M, Filipits M (2013). HCRP1 expression status is a significant prognostic marker in oral and oropharyngeal cancer. Oral Dis.

[41] Xu J, Zhang X, Wang H, Ge S, Gao T, Song L, Wang X, Li H, Qin Y, Zhang Z (2017). HCRP1 downregulation promotes hepatocellular carcinoma cell migration and invasion through the induction of EGFR activation and epithelial-mesenchymal transition. Biomed Pharmacother.

[42] Yang W, Wang JG, Xu J, Zhou D, Ren K, Hou C, Chen L, Liu X (2017). HCRP1 inhibits TGF-beta induced epithelial-mesenchymal transition in hepatocellular carcinoma. Int J Oncol.

[43] Yang W, Wang JG, Wang Q, Qin Y, Lin X, Zhou D, Ren K, Hou C, Xu J, Liu X (2016). Decreased HCRP1 promotes breast cancer metastasis by enhancing EGFR phosphorylation. Biochem Biophys Res Commun.

[44] Xu L, Ding Y, Catalona WJ, Yang XJ, Anderson WF, Jovanovic B, Wellman K, Killmer J, Huang X, Scheidt KA, Montgomery RB, Bergan RC (2009). MEK4 function, genistein treatment, and invasion of human prostate cancer cells. J Natl Cancer Inst.

