# Supplementary material for: MicroRNA-19a acts as a prognostic marker and promotes prostate cancer progression via inhibiting VPS37A expression
Source: Oncotarget. 2017 Dec 6;9(2):1931–43. doi: 10.18632/oncotarget.23026 (PMC5788610; doi:10.18632/oncotarget.23026)
Supplement: Supplementary file 1 [file oncotarget-09-1931-s001.pdf]

MicroRNA-19a acts as a prognostic marker and promotes prostate cancer progression via inhibiting VPS37A expression

SUPPLEMENTARY MATERIALS

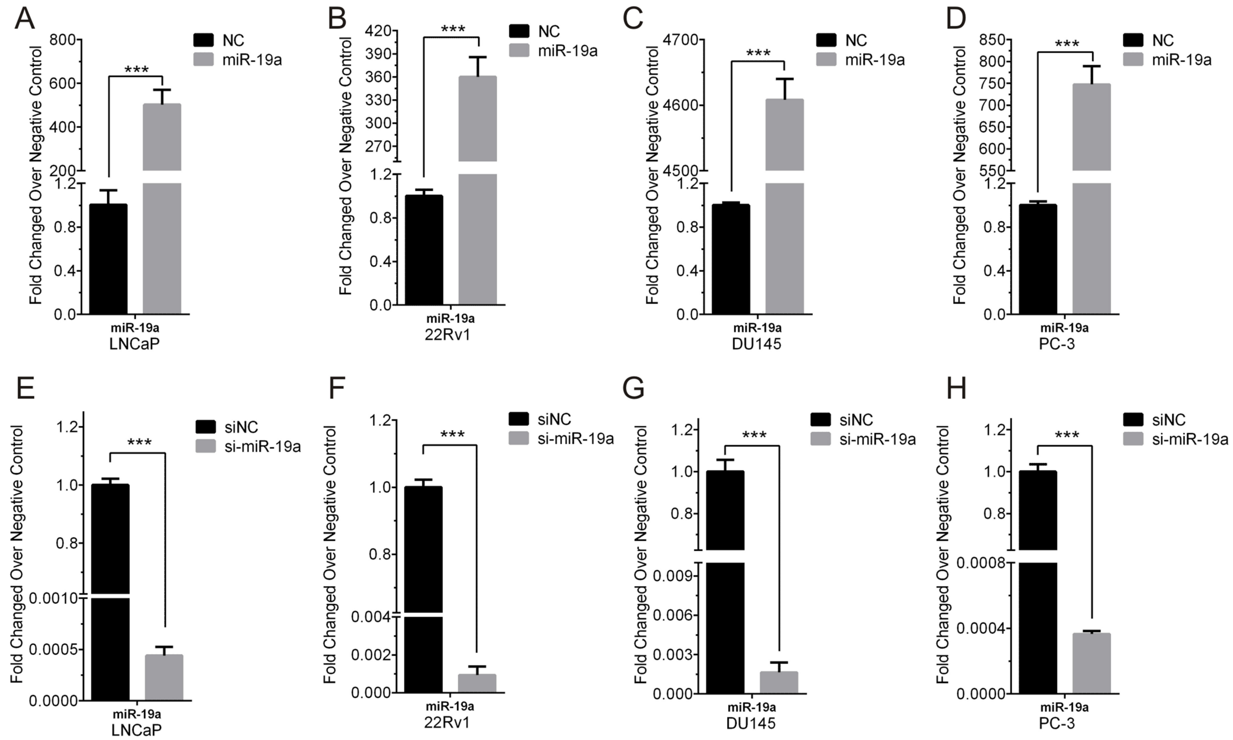

Supplementary Figure 1: The transfection efficiency of miR-19a mimics and inhibitor in PCa cells. (**A–D**) qRT-PCR of miR-19a after miR-19a mimics transfection in LNCaP (A), 22Rv1 (B), DU145 (C) and PC-3 (D) as indicated. RNU6 was used as internal control. (**E–H**) qRT-PCR of miR-19a after miR-19a inhibitor transfection in LNCaP (E), 22Rv1 (F), DU145 (G) and PC-3 (H) as indicated. RNU6 was used as internal control. Significance was defined as $P < 0.05$ ($^{*}P < 0.05$; $^{**}P < 0.01$; $^{***}P < 0.001$); ns means not significant.

Supplementary Table 1: Expression of up-regulated miRNAs in prostate cancer versus normal tissue samples

MiRNA Name	TCGA			GSE21306			GSE76260		
	***P*-value**	**T**	***N***	***P*-value**	**T**	***N***	***P*-value**	**T**	***N***
hsa-miR-32	1.60E-05	37.45	20.43	2.74E-08	6.24	5.15	2.75E-04	8.81	7.62
hsa-miR-19a	1.29E-07	21.82	8.03	5.31E-06	9.90	9.25	1.18E-02	12.74	11.84
hsa-miR-18b	6.58E-03	1.34	0.59	1.50E-03	5.59	5.15	2.26E-02	10.77	10.22
hsa-miR-96	1.33E-14	29.74	5.84	8.36E-13	9.27	7.68	2.12E-02	11.28	10.74
hsa-miR-183	6.73E-14	5970.82	1354.36	1.77E-14	6.63	4.86	1.52E-03	11.82	11.26
hsa-miR-130b	4.77E-06	11.75	5.03	1.15E-06	6.86	6.15	4.71E-03	10.64	10.18
hsa-miR-182	2.08E-18	20411.6	3831.3	1.82E-11	5.61	4.56	7.21E-03	12.41	12.03
hsa-miR-18a	1.38E-05	5.24	2.09	5.57E-07	5.33	4.65	2.50E-03	12.41	12.04
hsa-miR-375	7.18E-23	107796	21077	2.64E-04	11.69	10.68	7.54E-04	14.27	13.90
hsa-miR-106a	5.05E-08	104.38	31.43	3.18E-02	6.77	6.54	1.93E-03	14.11	13.86
hsa-miR-106b	1.68E-12	298.03	164.07	2.22E-09	10.44	9.80	7.71E-03	13.27	13.05
hsa-miR-425	1.21E-09	110.23	37.05	1.08E-06	7.82	7.23	1.04E-02	12.77	12.62
hsa-miR-17	2.45E-14	596.34	216.81	4.88E-05	10.23	9.66	3.93E-03	14.09	13.93
hsa-miR-25	1.32E-23	6548.03	2348.38	1.08E-11	9.68	8.88	1.34E-03	14.14	14.00
hsa-miR-93	1.11E-16	3730.10	835.69	9.46E-10	9.37	8.65	1.62E-04	14.05	13.94
hsa-miR-200c	2.37E-22	13004.0	3339.58	1.95E-06	12.01	11.20	3.89E-02	14.61	14.53

T represents PCa tumor tissues and N represents normal prostate tissues. For each miRNA, *P*-value and the relative miRNA expression level in tumor and normal tissues are presented in the table.

Supplementary Table 2: Expression of down-regulated miRNAs in prostate cancer versus normal tissue samples

MiRNA Name	TCGA			GSE21306			GSE76260		
	***P*-value**	**T**	***N***	***P*-value**	**T**	***N***	***P*-value**	**T**	***N***
hsa-miR-100	1.60E-03	8015.7	9402.1	3.16E-04	10.69	11.42	4.22E-02	14.39	14.42
hsa-miR-23a	7.47E-05	1338.6	1712.0	6.94E-04	12.35	12.86	2.08E-02	14.09	14.21
hsa-miR-29a	3.34E-02	5970.0	6560.9	6.94E-04	12.35	12.86	3.47E-03	13.79	13.93
hsa-miR-23b	4.08E-12	1460.4	2140.2	4.04E-06	12.62	13.49	1.10E-03	14.20	14.34
hsa-miR-27b	9.36E-21	1562.8	2381.0	5.38E-07	12.04	12.93	3.91E-04	14.21	14.36
hsa-miR-378	2.51E-02	375.23	501.44	3.54E-11	7.06	7.96	3.87E-02	13.22	13.37
hsa-miR-221	2.18E-28	191.54	422.50	1.71E-10	9.10	10.64	9.60E-04	14.29	14.49
hsa-miR-222	1.05E-12	48.65	85.11	1.67E-11	8.41	10.11	2.42E-04	13.78	14.05
hsa-miR-152	9.73E-19	226.09	340.58	1.67E-08	7.05	7.80	1.88E-05	13.66	13.98
hsa-miR-205	1.03E-04	826.95	1357.2	5.35E-05	9.71	12.60	3.46E-02	13.86	14.39
hsa-miR-379	5.37E-09	439.11	624.78	3.56E-02	4.10	4.35	4.60E-02	9.25	9.71
hsa-miR-136	5.65E-09	32.66	55.39	9.05E-03	7.27	7.66	2.45E-02	9.39	9.86
hsa-miR-452	1.61E-09	34.71	48.77	1.94E-09	5.01	5.79	1.67E-05	9.02	9.83

T represents for PCa tumor tissues and N represents for normal prostate tissues. For each miRNA, *P*-value and the relative miRNA expression level in tumor and normal tissues are presented in the table.

Supplementary Table 3: Primers used for RT-PCR, clone, mutation and transfection

Name	Forward primer (5′-3′)	Reverse primer (5′-3′)
β-ACTIN	CCTCTCCCAAGTCCACACAG	GGGCACGAAGGCTCATCATT
RNU6	CGCTTCGGCAGCACATATACTAA	TATGGAACGCTTCACGAATTTGC
VPS37A	CTTCAGCAGGCGATAGCAA	CTCTTGGCAGTTCATGTTTCC
3'UTR VPS37A	GCACATGAAGCTGAGGAAGAATCTG	GTGAGATGGCACATAGGCAGGTT
3'UTR VPS37A-mut	GCACTACAGTAGATGAAATGTTT ATTTATTGTTGATAAATTGTATC	ATTTCATCTACTGTAGTGCAGAAA AATTTCTCAAAGATC
miRNA-NC	UUCUCCGAACGUGUCACGUTT	ACGUGACACGUUCGAGAATT
has-miR-19a mimics	UGUGCAAAUCUAUGCAAAACUGA	AGUUUUGCAUAGAUUUGCACAUU
miRNA inhibitor-NC	CAGUACUUUUGUGUAGUACAA	
hsa-miR-19a inhibitor	UCAGUUUUGCAUAGAUUUGCACA	
